# Application of PLA/GO/ZnO and PLA/GO/Cu_2_O as sensor

**DOI:** 10.1038/s41598-024-65913-5

**Published:** 2024-09-25

**Authors:** Khaled S. Amin, Mohamed M. Yassin, Yahia M. Abdallah, Yusuf M. Alsayyad, Hanan Elhaes, Medhat A. Ibrahim

**Affiliations:** 1 Physics Department, Faculty of Science, Al-Azhar University, Nasr City, Cairo, 11884 Egypt; 2 Physics Department, Faculty of Women for Arts, Science and Education, Ain Shams University, Cairo, 11757 Egypt; 3 Spectroscopy Department, National Research Centre, 33 El-Bohouth St., Dokki, 12622 Giza Egypt; 4 Molecular Modeling and Spectroscopy Laboratory, Centre of Excellence for Advanced Science, National Research Centre, 33 El-Bohouth St., Dokki, 12622 Giza Egypt

**Keywords:** PLA, GO, DFT: B3LYP/LANL2DZ, Gas senor and metal oxide semiconductors, Environmental sciences, Materials science

## Abstract

Polylactic acid modified with graphene oxide (PLA/GO) is proposed to interact with ZnO through 6 different schemes. Density functional theory at B3LYP/LANL2DZ level was utilized to calculate total dipole moment (TDM), HOMO/LUMO energy gap (ΔE) and to map the molecular electrostatic potential (MESP). Results indicated that PLA/GO interacted with ZnO through O-atom forming PLA/GO/OZn composite. This composite interacts with methane, hydrogen sulfide, humidity (H_2_O), carbon dioxide and ethanol. The same gases were supposed to interact further with PLA/GO/Cu_2_O. Adsorption energy for the interaction between each composite and the proposed gases were calculated. Both PLA/GO/OZn and PLA/GO/Cu_2_O composites interacted favorably with H_2_O. Adsorption energy for interaction of other gases with studied structures are generally low compared to H_2_O. PLA/GO/OZn have adsorption energy slightly higher than that of PLA/GO/Cu_2_O. PLA/GO/OZn has higher TDM values than those of PLA/GO/Cu_2_O, indicating a more polar material. Conversely, PLA/GO/Cu_2_O exhibited larger ΔE values than those of PLA/GO/OZn. TDM and energy gap results for both studied structures indicated good sensing capabilities. Further insights come from analyzing the calculated density of states (DOS) and partial density of states (PDOS). PLA/GO/Cu_2_O exhibited high peak for copper in its DOS and PDOS spectra compared to zinc and oxygen in case of PLA/GO/OZn. This means a higher density of available electronic states associated with Cu.

## Introduction

Polylactic acid (PLA) is a widely explored polymer for various applications due to its biodegradability and eco-friendly characteristics. Despite its potential, PLA faces challenges such as brittleness and poor thermal stability. To address these limitations, innovations in renewable plasticizers and nanocomposites have been pursued^1–5^. Blending this important polymer with other featured materials could improve and/or overcome its limitations. Graphene oxide (GO) is the oxidized form of graphene, characterized as the enhanced form of graphene with high surface area and excellent thermal stability^6,7^. GO surface is functionalized with some oxygen-based functional groups, hydroxyl (–OH), alkoxy (C–O–C), carbonyl (C=O), carboxyl (–COOH)^8^. GO possesses unique physical, chemical and mechanical properties according to its functionalization which dedicate this member of graphene family for many applications^9–11^. One strategy involves integrating GO into PLA, forming PLA/GO nanocomposites, which exhibit enhanced mechanical strength, thermal stability, and other properties. Additionally, semiconductor metal oxide-based materials, such as zinc oxide (ZnO), are being investigated for gas sensing applications due to their unique properties^12–15^. ZnO shows potential application as gas sensor for many gases such as ammonia^16^. ZnO shows also excellent selectivity and high response to NO_2_^17^. ZnO nanoparticles under UV acts as sensor for CO_2_, the sensor was activated at relatively low temperatures < 100 °C^18^. ZnO also could be effectively used as senor for volatile organic compounds^19,20^. It is reported that ZnO acted as sensor after heating in order to activate its surface^21^. It was also reported that the performance of ZnO sensors could be improved. A hybrid approach involving GO/ZnO on a PLA substrate is proposed by combining theoretical insights with experimental techniques. In this sense, some authors reported studies aiming to design and fabricate hybrid ZnO/GO nanocomposites on PLA substrates for efficient gas sensing applications^22–26^. On the other hand, many properties could be achieved by computational methods specially those depending on quantum mechanical calculations. Previous computational studies dedicated density functional theory (DFT) method for investigating graphene for bio-electronic applications^27^. Computational method at DFT was conducted to study the possible removal of organic pollutants such as Atrazine using graphene quantum dots^28^. Graphene was modified with some polymers including polyaniline/polyvinylidene fluoride/polytetrafluoroethylene, to enhance its application as electrode material for several electronic applications^29^. Both theoretical and experimental findings were conducted to design and implement carbon nitride modified with graphene to act as sensor for humidity^30^. DFT improved the ability of enhanced graphene to act as sensor, DFT was used to study the interactions between Fe_3_O_4_ and graphene clusters to model the nZVI/rGO substrate, as well as gold sheets and AsH_3_ molecules to simulate the sensor surface and analyte^31^.

The present work is conducted to apply DFT:B3LYP/LANL2DZ to study the possible interaction between PLA/GO/ZnO or PLA/GO/Cu_2_O with methane, hydrogen sulfide, humidity, carbon dioxide and ethanol respectively. Total dipole moment (TDM) and the difference between highest occupied molecular orbital (HOMO) and the lowest unoccupied molecular orbital (LUMO) (HOMO/LUMO energy gap (∆E)) will be calculated. Molecular electrostatic potential (MESP) and density of states (DOS), and partial density of states (PDOS) will be mapped. Through this synergistic approach, the goal is to develop advanced nanocomposites based on polymeric material and metal oxide with superior properties, particularly in gas sensing, by leveraging the strengths of PLA, GO, and ZnO.

### Calculation details

All the studied structures were subjected to calculations using Gaussian 09 program^32^ implemented at Molecular Modeling and Spectroscopy Laboratory, Centre of Excellence for Advanced Science, NRC, Egypt. Each structure is calculated with density functional theory DFT at Becke’s three-parameter exchange functional in conjunction with the Lee–Yang–Parr correlation functional B3LYP^33–35^ using Los Alamos National Laboratory 2 double ζ LANL2DZ basis set which is used for Zn atom.

The total dipole moment TDM and the HOMO/LUMO energy band gap (∆E) were calculated at the same level of theory.

The adsorption energy E_a_ for the system of the composite interacting with gas could be calculated from the following equation: E_a_ =  − [E_system_ − (E_adsorbent_ + E_adsorbate_)].

To assess the active sites on surfaces, the molecular electrostatic potential MESP was mapped. For better understanding of electronic properties both density of stated DOS and partial density of states PDOS were mapped also at the same level of theory.

The error within the computational method is systematic while other level of theory was consulted for comparison. At DFT level both 6-31g(d,p) will be compared with LANL2DZ, also WB97XD is compared with B3LYP.

Some reactivity descriptors are calculated such as ionization potential (I), electronic chemical potential (µ) and chemical hardness (η) to describe the reactivity of the studied molecules^36^. Whereas, these quantities are derived from the following equations

1 $$I=-{E}_{HOMO},$$

2 $$A=-{E}_{LUMO},$$

3 $$\mu =\frac{I+A}{2},$$

4 $$\eta =\frac{I-A}{2}.$$

## Results and discussion

Before conducting the molecular modeling calculations, the first step is to describe how the studied model molecules were designed.

### Building model molecules

**Figure 1:**
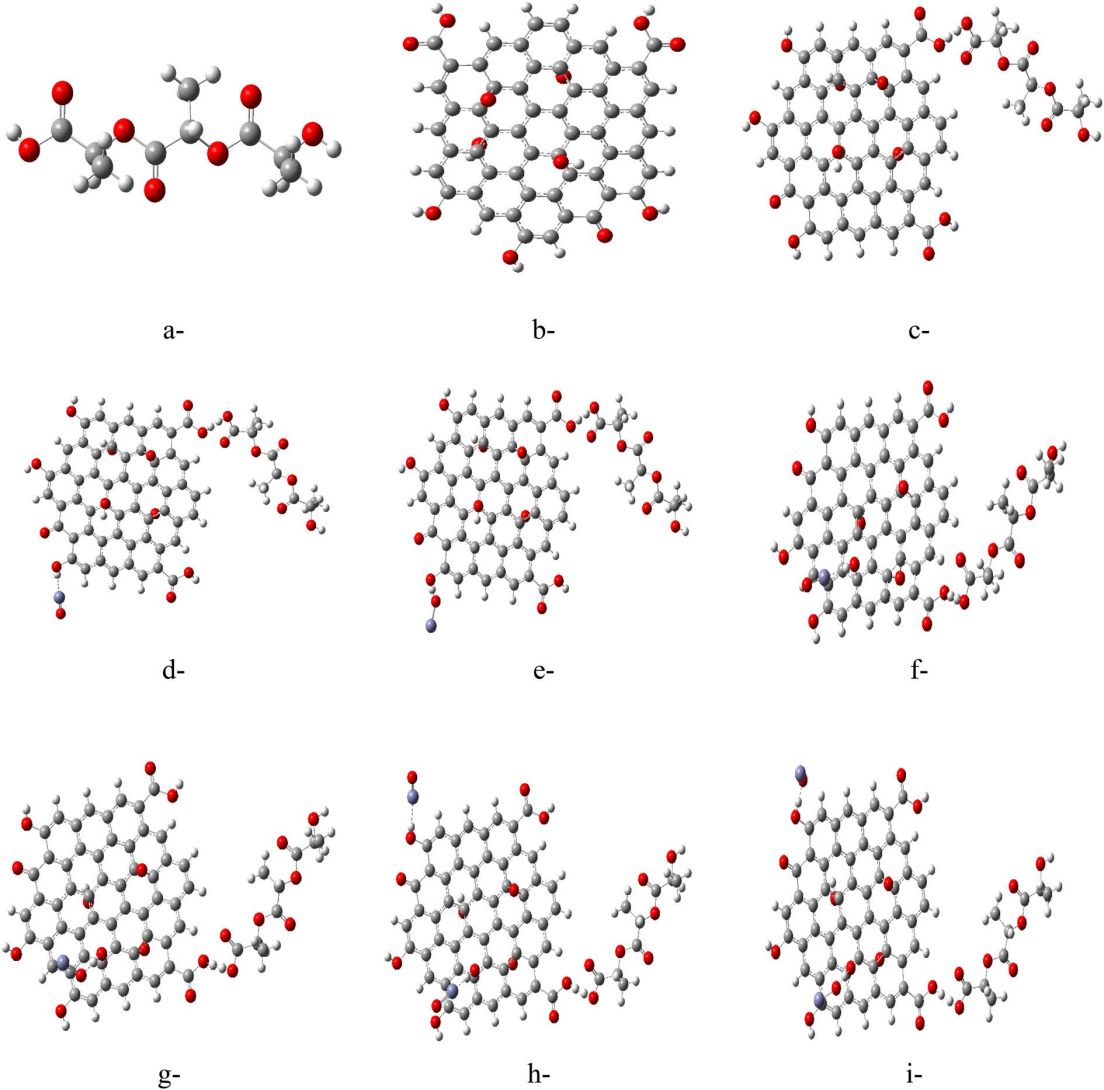
Studied model molecules for, (**a**) PLA, (**b**) GO, (**c**) PLA/GO composite, (**d**) PLA/GO/ZnO (Terminal OH), (**e**) PLA/GO/OZn (Terminal OH), (**f**) PLA/GO/ZnO (Inner OH), (**g**) PLA/GO/OZn (Inner OH), (**h**) PLA/GO/2ZnO (Terminal and Inner OH) and (**i**) PLA/GO/2OZn (Terminal and Inner OH).

The electronic charge transport for metal oxide semiconductor (MOS) dedicates them for several applications. The MOS have valence described with ionic bonding. Electron transport in MOS coming from the 2p orbital of oxygen plays an important role beside the existence in metals valence band^37^. A model of 3 units PLA polymer chain is used to interact with GO and consequently PLA/GO interact with ZnO from different positions. The PLA was modified with GO then metal oxide according to the following schemes, indicated in Fig. 1, showing 9 model molecules that were designed for this work. Figure 1a shows the PLA model structure which consists of three units. Figure 1b indicates the model of GO, which is functionalized with two carboxyl groups COOH, two epoxides O and five OH groups. The next model is PLA/GO composite whereas PLA was interacted weakly through OH of COOH located in GO as shown in Fig. 1c. PLA/GO composite is supposed to weakly interact with ZnO through terminal OH of GO and Zn of ZnO as indicated in Fig. 1d, forming PLA/GO/ZnO (Terminal OH). Figure 1e presented the possible interaction of ZnO with PLA/GO composite through the O of ZnO forming PLA/GO/OZn (Terminal OH). Figure 1f shows a model molecule for PLA/GO/ZnO (Inner OH) where in this model the ZnO interacted with the inner OH of GO. Figure 1g represents the same model as in Fig. 1f but in this case ZnO is interacting throughout O forming PLA/GO/OZn (Inner OH). Figure 1h presented the interaction of PLA/GO with two ZnO through Zn atoms, where one ZnO interacted with terminal OH of GO, while the other interacting with inner OH of GO forming PLA/GO/2ZnO (Terminal and Inner OH). Finally, the model for PLA/Go composite interacting with two ZnO through O atoms, one ZnO interacting with terminal OH of GO while the other interacting with inner OH of GO forming PLA/GO/2OZn (Terminal and Inner OH) is indicated in Fig. 1i.

### Calculated physical parameters

Two physical descriptors were calculated; namely TDM and ∆E. Table 1 presented B3LYP/LANL2DZ calculated TDM as Debye and ∆E as eV for the studied model molecules. It was stated earlier that these two descriptors gave a good indication for the reactivity of the studied structure^38^. Increasing TDM with decreasing ∆E indicates the ability of the structure to interact with its surrounding molecules. The high the TDM with corresponding lower ∆E is an indication for the reactivity of the studied compound^39,40^. As indicated in Table 1, the TDM of PLA was 3.879 Debye, while ∆E was 5.933 eV. As PLA interacts with GO and ZnO, TDM increased to reach 12.518 Debye for PLA/GO/2ZnO (Terminal and Inner OH), while ∆E decreased to reach 0.236 eV for PLA/GO/2OZn (Terminal and Inner OH), whereas this model structure still has high TDM (9.591 Debye). These results are an indication that the interactions enhanced the electrical characteristics of the proposed model molecules.

**Table 1:**
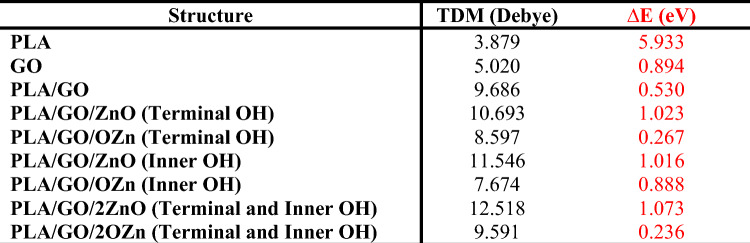
B3LYP/LANL2DZ calculated TDM as Debye and ∆E as eV for the studied model molecules.

### Mapping molecular electrostatic potential MESP

The MESP is an important parameter which describes the reactivity of the studied surfaces. The reactivity of the surface is indicated in terms of a color scheme, where the distribution of charges could be indicated on the surface by mapping the colors which can be ordered as Red > Orange > Yellow > Green > Blue, such that the red color on the MESP surface representing the highest charge zone, the blue representing the lowest charge zone and the yellow is the middle and/or neutral charge zone^39^.

**Figure 2:**
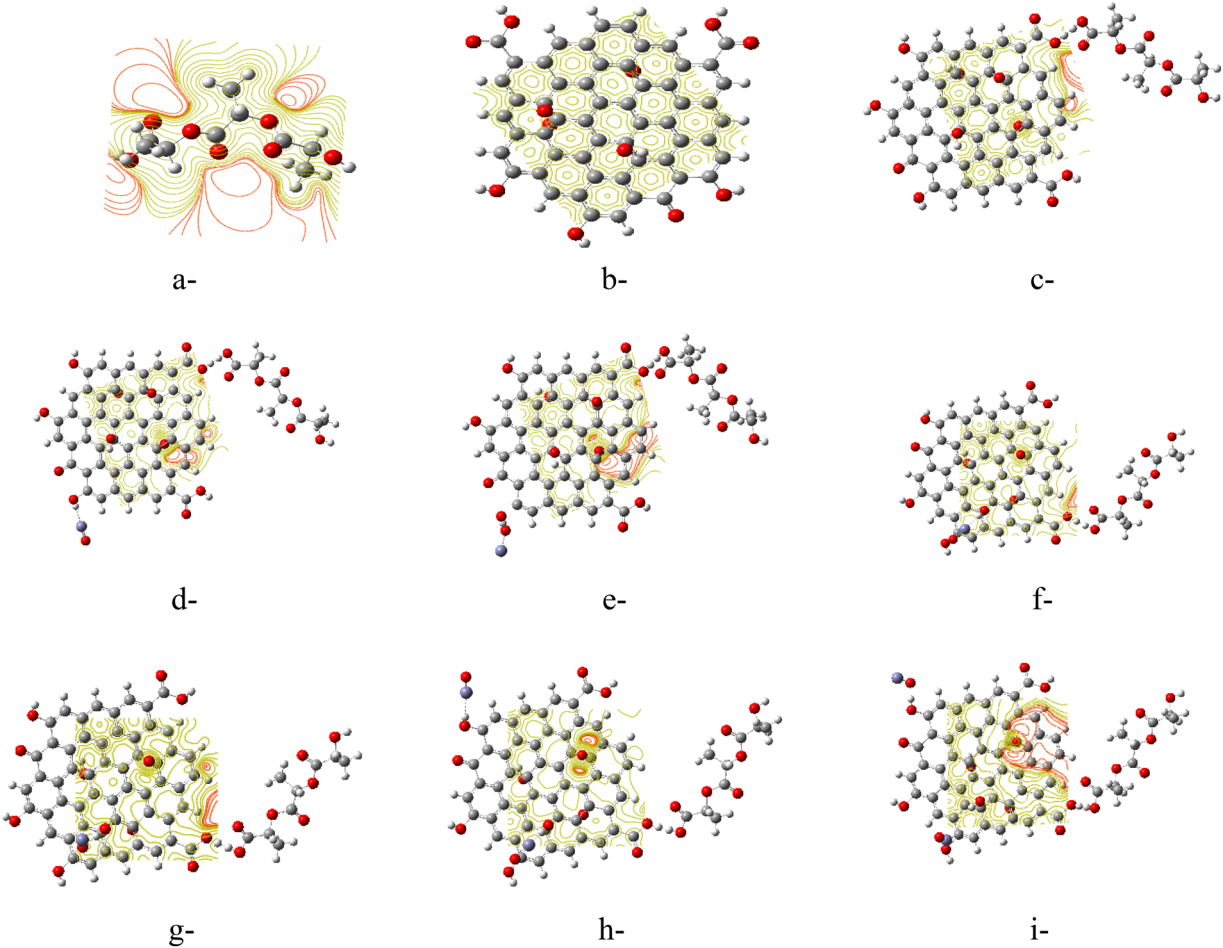
B3LYP/LANL2DZ calculated MESP for the studied model molecules whereas, (**a**) PLA, (**b**) GO, (**c**) PLA/GO composite, (**d**) PLA/GO/ZnO (Terminal OH), (**e**) PLA/GO/OZn (Terminal OH), (**f**) PLA/GO/ZnO (Inner OH), (**g**) PLA/GO/OZn (Inner OH), (**h**) PLA/GO/2ZnO (Terminal and Inner OH) and (**i**) PLA/GO/2OZn (Terminal and Inner OH).

Figure 2 shows the MESP map for all studied model molecules. Figure 2a shows the MESP map for PLA with reactive potential around oxygen in terms of the red color, while GO shows neutral potential on its surface in terms of the yellow color as shown in Fig. 2b. As far as PLA interacted with GO, the surface edge of GO became more reactive as the surface colored with intermediate colors between orange and yellow, as indicated in Fig. 2c. Figure 2d–i represent MESP maps for the interaction of PLA/GO with ZnO for the different positions of interaction. When PLA/GO interacted with ZnO through Zn and the interaction took place through terminal OH of graphene, low- red color was localized mainly around the edge oxygen atom of GO. The same behavior is noticed for other composites where the reactivity on the surface of GO was close to the edge interacting with PLA. These obtained MESP results are in good agreement with TDM and ΔE results. As a result, the electronic properties of PLA/GO with ZnO were improved and may be employed in different applications, especially as a sensor based on their reactive surface.

### Sensing of gases

**Figure 3:**
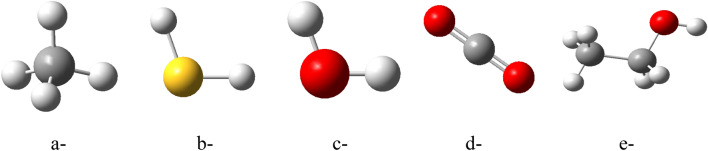
Studied model molecules for (**a**) CH_4_, (**b**) H_2_S, (**c**) H_2_O, (**d**) CO_2_ and (**e**) C_2_H_5_OH.

Based on the results of TDM and ∆E for the studied structures, PLA/GO/OZn (Terminal OH) showed the most reactive structure. Therefore, PLA/GO/OZn (Terminal OH) is dedicated in this work to interact with some gases as well as a volatile organic compound to test its ability for gas sensing. Figure 3 presents the smallest proposed model for Methane (CH_4_), Hydrogen Sulfide (H_2_S), Humidity (H_2_O), Carbon Dioxide (CO_2_) and Ethanol (C_2_H_5_OH), which is studied as an example for volatile organic compound.

#### Interaction between PLA/GO/OZn and gases

**Figure 4:**
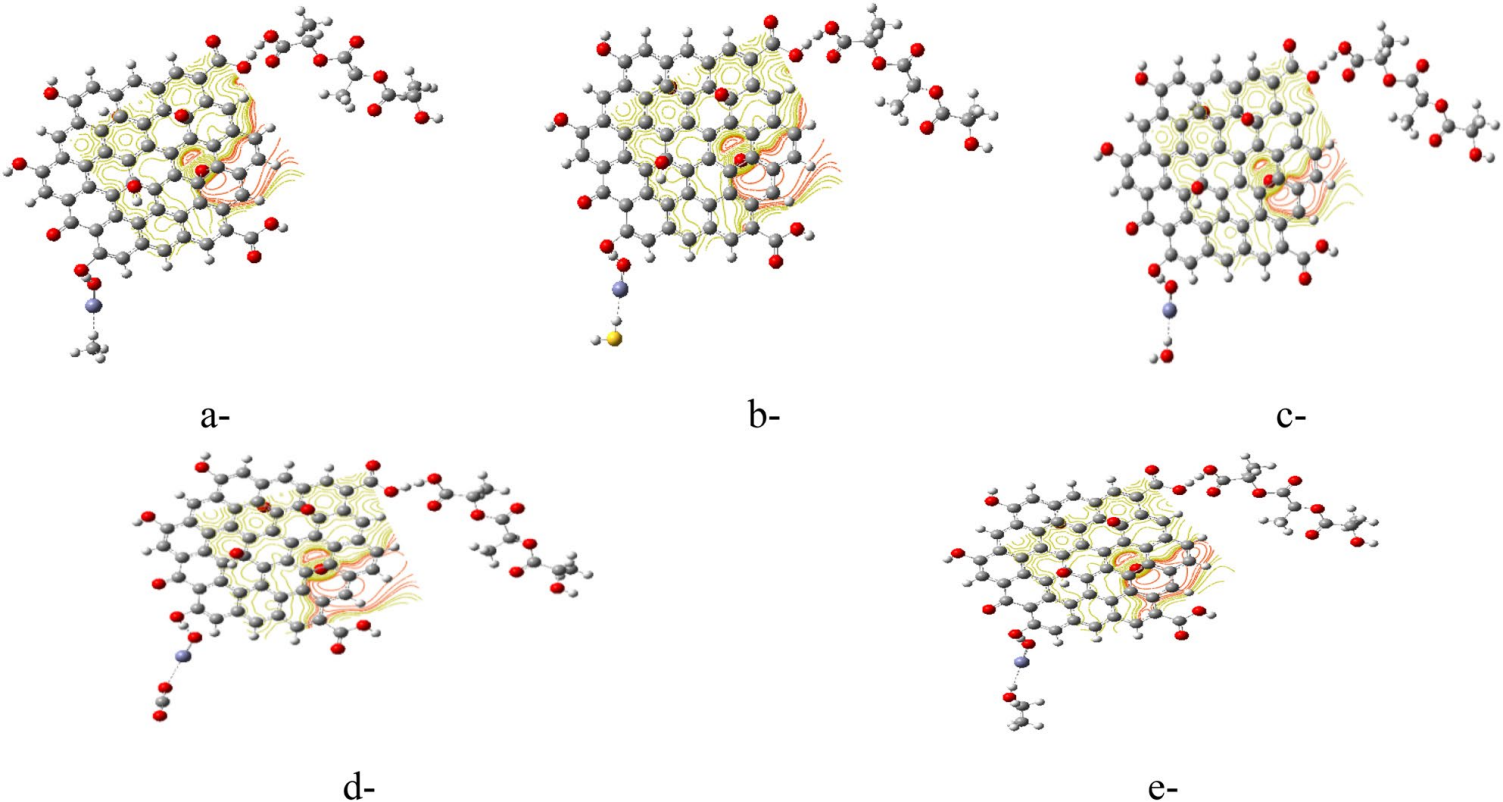
B3LYP/LANL2DZ calculated MESP for the PLA/GO/OZn (Terminal OH) interacted with (**a**) CH_4_, (**b**) H_2_S, (**c**) H_2_O, (**d**) CO_2_ and (**e**) C_2_H_5_OH.

Figure 4 presented the calculated MESP map for the PLA/GO/OZn (Terminal OH) interacted with CH_4_, H_2_S, H_2_O, CO_2_ and C_2_H_5_OH. From the MESP maps as indicated in the Fig. 4a–e, the PLA/GO/OZn (Terminal OH) remains active after interactions with the gases.

Table 2 presents the calculated TDM and ΔE for the PLA/GO/OZn (Terminal OH) interacting with the studied gas molecules. TDM and ΔE were found to be 8.597 Debye and 0.267 eV, respectively, for PLA/GO/OZn (Terminal OH).

**Table 2:** B3LYP/LANL2DZ calculated TDM as Debye and ∆E as eV for the PLA/GO/OZn (Terminal OH) interacting with different gases.

Structure	TDM (Debye)	∆E (eV)
PLA/GO/OZn	8.597	0.267
PLA/GO/OZn/CH_4_	12.418	0.801
PLA/GO/OZn/H_2_S	10.892	0.262
PLA/GO/OZn/H_2_O	6.891	0.245
PLA/GO/OZn/CO_2_	10.227	0.510
PLA/GO/OZn/C_2_H_5_OH	8.350	0.350

As the gases interacted with PLA/GO/OZn (Terminal OH), the TDM increased from 8.597 Debye to 12.418, 10.892 and 10.227 Debye for CH_4_, H_2_S, and CO_2_ respectively. A slight decrease in TDM was observed as PLA/GO/OZn (Terminal OH) interacted with C_2_H_5_OH (8.350 Debye) and H_2_O (6.891 Debye), although they still had high values of TDM, which means that they remained active. Despite the higher increase in the TDM of PLA/GO/OZn/CH_4_, its ΔE increased from 0.267 eV for PLA/GO/OZn to 0.801 eV for PLA/GO/OZn/CH_4_. In contrast for PLA/GO/OZn/H_2_O, TDM decreased but its ΔE also decreased to 0.245 eV which is the lowest value of the studied structures.

Combining these results with the mapping of the MESP, one can conclude that the surface of the studied PLA/GO/OZn remained active after interaction with gases which is an indication for its ability to interact further.

#### Interaction between PLA/GO/Cu_2_O and sensing of gases

Table 3 presents the calculated TDM and ∆E for the PLA/GO/Cu_2_O (Terminal OH) interacting with the studied gas molecules. PLA/GO/Cu_2_O showed TDM of 5.000 Debye while ∆E was 1.152 eV. For adsorption of CH_4_, TDM was 4.873 Debye, while ∆E was 0.646 eV. And for adsorption of H_2_O, the TDM was 6.293 Debye, while ∆E was 0.554 eV. Higher TDM of 10.650 Debye was regarded corresponding to the adsorption of CO_2_, but the ∆E was 1.038 eV, yet still lower than PLA/GO/Cu_2_O (Terminal OH). From these results, the interaction of PLA/GO/Cu_2_O with H_2_O showed the lowest value of ∆E. All the studied structures remained active after interaction with gases.

**Table 3:** B3LYP/LANL2DZ calculated TDM as Debye and ∆E as eV for the PLA/GO/Cu_2_O interacting with different gases.

Structure	TDM (Debye)	∆E (eV)
PLA/GO/Cu_2_O	5.000	1.152
PLA/GO/Cu_2_O/CH_4_	4.873	0.646
PLA/GO/Cu_2_O/H_2_S	5.173	0.557
PLA/GO/Cu_2_O/H_2_O	6.293	0.554
PLA/GO/Cu_2_O/CO_2_	10.650	1.038
PLA/GO/Cu_2_O/C_2_H_5_OH	7.241	0.652

**Figure 5:**
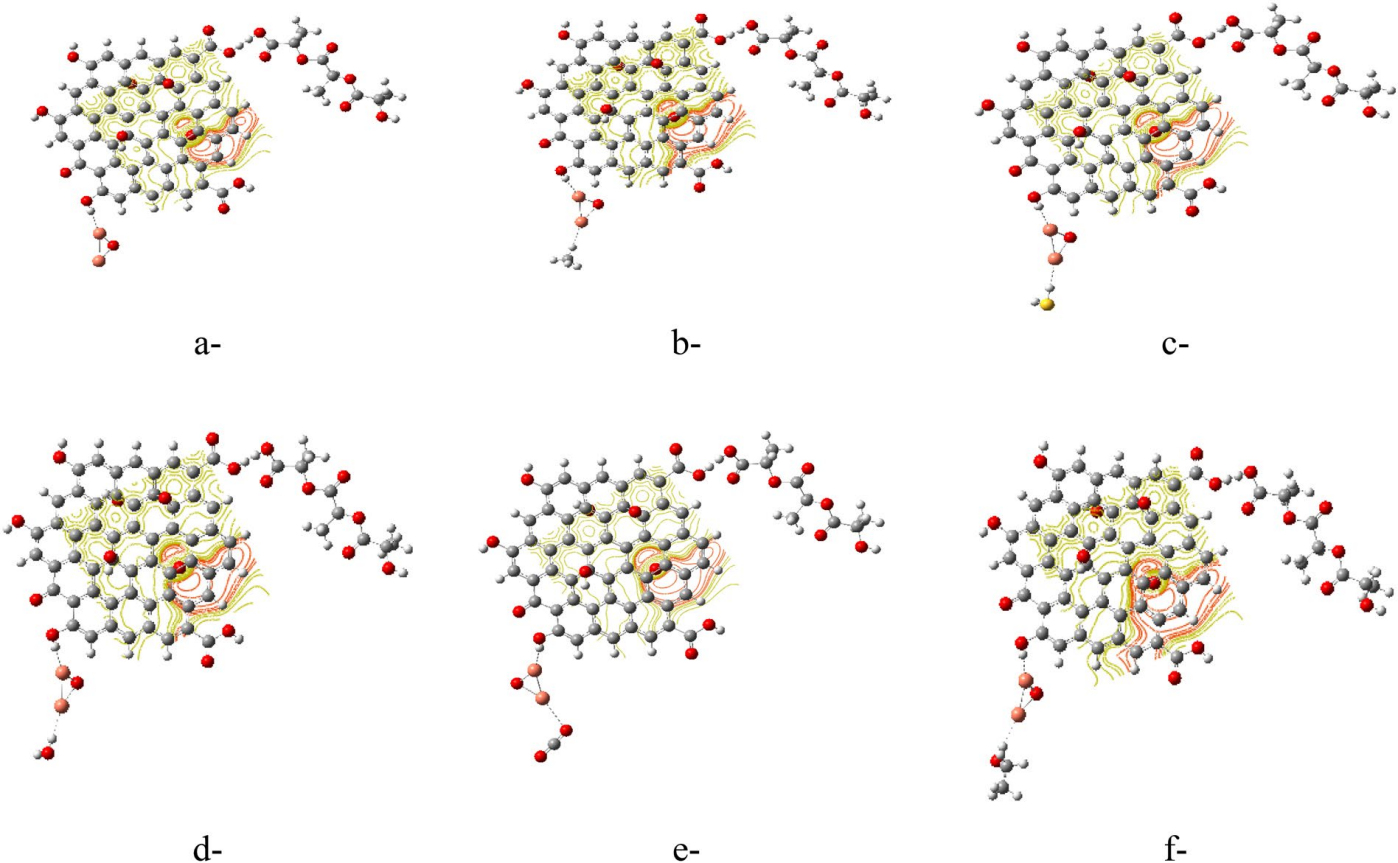
B3LYP/LANL2DZ calculated MESP for (**a**) PLA/GO/Cu_2_O and PLA/GO/Cu_2_O interacted with (**b**) CH_4_, (**c**) H_2_S, (**d**) H_2_O, (**e**) CO_2_, (**f**) C_2_H_5_OH.

Another important surface parameter is to map the MESP of the PLA/GO/Cu_2_O after interacting with the studied gases as indicated in Fig. 5. As seen in case of the PLA/GO/OZn, the surface of the PLA/GO/Cu_2_O remains active as indicated by the calculated MESP map. Therefore, the PLA/GO/Cu_2_O could further interact and accept adsorption of the studied gases.

#### Calculating the adsorption energy

One important step to indicate the selectivity of the studied composites to the interacting gases is to calculate the adsorption energy. The adsorption energy E_a_ for the system of the composite interacting with gas could be calculated and listed in Table 4. The adsorption energy which listed in Table 4, comes as the studied gases adsorbed onto PLA/GO/OZn (Terminal OH) and PLA/GO/Cu_2_O (Terminal OH). Each gas of the studied four gases namely CH_4_, H_2_S, H_2_O, CO_2_ and the volatile organic compound C_2_H_5_OH is supposed to interact separately with PLA/GO/OZn and then with PLA/GO/Cu_2_O.

**Table 4:** Calculated adsorption energy of the studied gases onto PLA/GO/OZn (Terminal OH) and PLA/GO/Cu_2_O (Terminal OH) at B3LYP/LANL2DZ.

Structure	Adsorption energy
PLA/GO/OZn	
PLA/GO/OZn/CH_4_	0.1081
PLA/GO/OZn/H_2_S	0.1030
PLA/GO/OZn/H_2_O	− 1.0410
PLA/GO/OZn/CO_2_	− 0.0200
PLA/GO/OZn/C_2_H_5_OH	0.0430
PLA/GO/Cu_2_O	
PLA/GO/Cu_2_O/CH_4_	0.0191
PLA/GO/Cu_2_O/H_2_S	− 0.0632
PLA/GO/Cu_2_O/H_2_O	− 1.1755
PLA/GO/Cu_2_O/CO_2_	0.0145
PLA/GO/Cu_2_O/C_2_H_5_OH	0.0529

Each gas (CH_4_, H_2_S, H_2_O, CO_2_, and C_2_H_5_OH) is supposed to interact separately with PLA/GO/OZn and then PLA/GO/Cu_2_O.

The calculated adsorption energies indicated both endothermic and exothermic processes. Based on the negative values of adsorption energy, we can see that (H_2_O) has the strongest favorable interaction with both PLA/GO/OZn and PLA/GO/Cu_2_O composite materials. This indicates that water molecules are spontaneously adsorbed on these surfaces, releasing energy in the process.

Compared to the other gases, PLA/GO/Cu_2_O showed a stronger preference for adsorption for all the listed molecules except C_2_H_5_OH. This suggests that PLA/GO/Cu_2_O might be a better material for capturing these specific gas molecules. The positive adsorption energy values for CH_4_, CO_2_, and C_2_H_5_OH on both composites indicate weak and unfavorable interactions. These gases are less likely to be adsorbed on the surfaces or may require additional energy input for adsorption.

PLA/GO/Cu_2_O showed a generally stronger adsorption preference for most molecules compared to PLA/GO/OZn. This suggests a potentially higher affinity of Cu_2_O for these gas molecules. However, both materials showed a significant preference for humidity adsorption, with PLA/GO/Cu_2_O having a slightly stronger binding (more negative adsorption energy).

#### Calculating the density of states

The PLA/GO composite is interacted with OZn then Cu_2_O forming two composites interacted with the studied five gases. One important parameter for the studied structures containing metals is the density of states which is termed DOS. It referred to the number of states with particular energy level in which the electrons are allowed to occupy. The DOS gives brief insight into the electronic as well as the optical properties of the studied structures. The number of quantum states of the electrons in the metal atom per unit volume per unit energy in the studied structures is known as the DOS. It could be also defined as the distribution of all obtainable quantum states per unit energy of molecule. Based on the electronic features in the DOS parameters, it is also known as continuous molecular property^41^. More precise insight could be achieved with partial density of states PDOS. It could be helpful to monitor the contributions of atoms into the occupied states.

**Figure 6:**
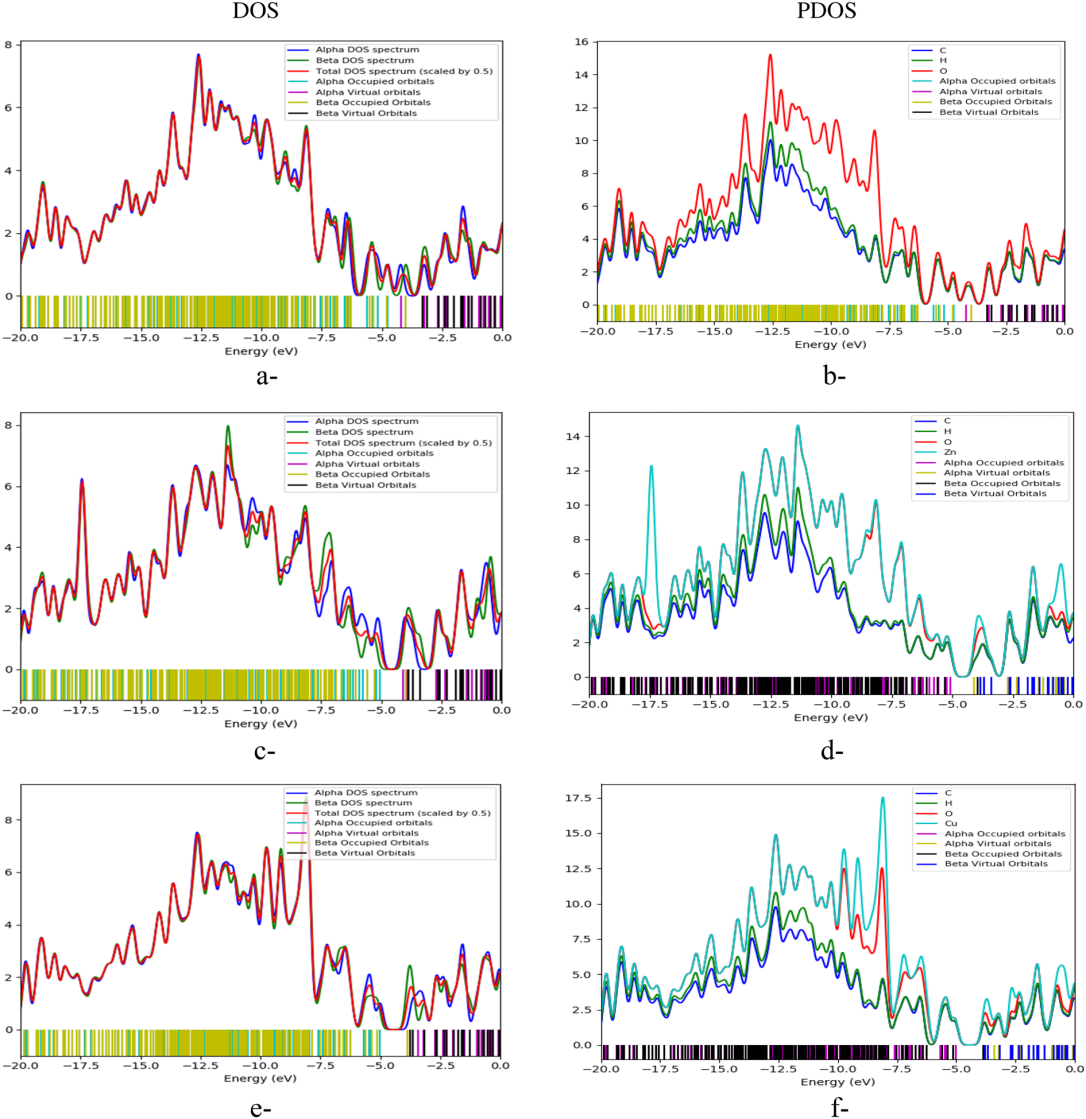
DOS and PDOS plots of model molecules for the structures (**a,b**) PLA/GO; (**c,d**) PLA/GO/OZn (Terminal OH); and (**e,f**) PLA/GO/Cu_2_O (Terminal OH).

Figure 6 presents the studied DOS and PDOS for the structures of PLA/GO (Fig. 6a,b), PLA/GO/OZn (Terminal OH) (Fig. 6c,d), and PLA/GO/Cu_2_O (Terminal OH) (Fig. 6e,f).

The DOS and PDOS curves show valence and conduction bands separated by small band gap. The valence band is mainly formed by oxygen’s 2p orbitals with a minor contribution from zinc’s 4s orbitals and the same for Cu_2_O with even more states which is shown by the higher peak. The bottom part of the conduction band of GO extends to the band gap of the ZnO and Cu_2_O. GO dominate the electronic conductibility of the composite^37^. Additionally, the noticeable shift towards the Fermi level indicates a favorable alteration in the band structure.

### Verification of the model

It is stated earlier that computational methods have systematic error which could be corrected with so called scale factor^42^. While scale factor is used for comparison with experimental results. As the present computational study contain no experimental results, another way for verification is conducted with the help of comparison with other level of theory. Table 5 presented the total dipole moment TDM as Debye and HOMO/LUMO band gap energy as eV which calculated at both B3LYP/LANL2DZ and B3LYP/6-31g(d,p) respectively. As indicated in Table 5, for PLA/GO/Cu_2_O both B3LYP/LANL2DZ and B3LYP/6-31g(d,p) show comparable results for calculated TDM and ∆E. While in case of PLA/GO/OZn, B3LYP/6-31g(d,p) model show slight higher TDM values with higher ∆E value. Comparing the results of both models, it is clear that both models show comparable results. Another verification of the model is tried as indicated in Table 6, in which TDM and ∆E were calculated at both WB97XD/6-31g(d,p) and B3LYP/6-31g(d,p) for PLA/GO/Cu_2_O structure. Results indicated comparable values for TDM as 4.251 Debye and 4.492 Debye while the ∆E was not comparable to each other.

**Table 5:** Total dipole moment TDM as Debye and HOMO/LUMO band gap energy as eV which calculated at both B3LYP/LANL2DZ and B3LYP/6-31g(d,p).

Structure	TDM (B3LYP)		∆E (B3LYP)	
	LANL2DZ	6-31g(d,p)	LANL2DZ	6-31g(d,p)
PLA/GO/Cu_2_O	5.000	4.492	1.152	1.168
PLA/GO/OZn	8.597	10.506	0.267	0.954

**Table 6:** Total dipole moment TDM as Debye and HOMO/LUMO band gap energy as eV which calculated at both WB97XD/6-31g(d,p) and B3LYP/6-31g(d,p) for PLA/GO/Cu_2_O structure.

Structure	TDM		∆E	
	WB97XD	B3LYP	WB97XD	B3LYP
PLA/GO/Cu_2_O	4.251	4.492	0.103	1.168

### Reactivity descriptors

Table 7 presented the reactivity descriptors which calculated at B3LYP/LANL2DZ level of theory. These descriptors include ionization potential, electronic chemical potential and chemical hardness. Ionization potential was 4.8570 eV for PLA/GO/OZn before the gases adsorbed into it. Slight decrease in the ionization potential was regarded as CH_4_, CO_2_ and C_2_H_5_OH adsorbed onto PLA/GO/OZn. While a slight increased regarded in case of both H_2_S and H_2_O. For PLA/GO/Cu_2_O the ionization potential was regarded as 5.006 eV the decreased as CH_4_ and C_2_H_5_OH interacted with PLA/GO/Cu_2_O. Other gases interacted with PLA/GO/Cu_2_O leads to a slight increase in ionization potential. Electronic chemical potential slightly decreased when the studied gases interacted with PLA/GO/OZn in contrast with PLA/GO/Cu_2_O which indicated an increased values of electronic chemical potential after gases interacted with PLA/GO/Cu_2_O.

**Table 7:** Reactivity descriptors, ionization potential (I) as eV, electronic chemical potential (μ) as eV and Chemical hardness (η) as eV which calculated at B3LYP/LANL2DZ level of theory.

Structure	Ionization potential (I)	Electronic chemical potential (μ)	Chemical hardness (η)
PLA/GO/OZn	4.8570	4.7233	0.1337
PLA/GO/OZn/CH_4_	4.7971	4.3150	0.4822
PLA/GO/OZn/H_2_S	4.8442	4.6184	0.2259
PLA/GO/OZn/H_2_O	5.0663	4.6240	0.4425
PLA/GO/OZn/CO_2_	4.7873	4.4375	0.3498
PLA/GO/OZn/C_2_H_5_OH	4.7003	4.4962	0.2041
PLA/GO/Cu_2_O	5.006	4.3298	0.6761
PLA/GO/Cu_2_O/CH_4_	4.8791	4.5411	0.3380
PLA/GO/Cu_2_O/H_2_S	5.0137	4.6584	0.3554
PLA/GO/Cu_2_O/H_2_O	5.0440	4.6834	0.3606
PLA/GO/Cu_2_O/CO_2_	5.1158	4.4808	0.6350
PLA/GO/Cu_2_O/C_2_H_5_OH	4.9422	4.6009	0.3412

Finally the chemical hardness for PLA/GO/OZn was 0.1337 then increased after the gases interact with PLA/GO/OZn. While it was 0.6761 for PLA/GO/Cu_2_O then decreased slightly after CO_2_ interacts PLA/GO/Cu_2_O with noticeable decreased corresponding to the remaining studied gases.

## Conclusion

DFT:B3LYP/LANL2DZ was used to model both PLA/GO/OZn and PLA/GO/Cu_2_O composites, then their possible interaction with 4 gases beside one volatile organic compound was also studied. Calculating the adsorption energy indicated selectivity of both composites for the adsorbed gases in terms of the studied physical parameters.

PLA/GO/OZn (ZnO-based) and PLA/GO/Cu_2_O (Cu_2_O-based) interacted with humidity more likely as compared with other gases. TDM and ΔE results indicated that both composites have the ability to interact with its surrounding molecules. PLA/GO/Cu_2_O showed TDM of about 5.000 Debye while ∆E was 1.152 eV. After adsorption of H_2_O, the TDM is 6.293 Debye, while ∆E was 0.554 eV.

Also, PLA/GO/Cu_2_O shows significant adsorption as compared with PLA/GO/OZn, which indicates potential higher affinity for the adsorbed gases. The most important remark is that both composites showed a significant preference for sensing humidity, with PLA/GO/Cu_2_O having stronger binding. The MESP calculation show that the surface edge became more reactive.

Calculated DOS and PDOS for PLA/GO/ZnO and PLA/GO/Cu_2_O indicated high density of the available electronic states. Moreover, the reactivity descriptors including ionization potential, electronic chemical potential and chemical hardness show a changes after PLA/GO/ZnO and PLA/GO/Cu_2_O interacted with the studied gases.

A change in the HOMO/LUMO energy comes from the fact that there is a change in the partial density of states. Although thers is no change in the ionization potential but other there are change in the total dipole moment, electronic chemical potential and chemical hardness. This may be due to partial changes in charges which makes the surface of the studied nanocomposites (PLA/GO/ZnO and PLA/GO/Cu_2_O) active and cable of interacting with gases and volatile organic compounds. The present computational model show comparable results for the same structure at WB97XD/6-31g(d,p) and B3LYP/6-31g(d,p) levels, especially for total dipole moment.

Based on the recorded changes in the studied physical parameters, the studied composites can be used as sensor, with selective behavior toward humidity as compared with other studied gases and moieties.

## Data Availability

The data that support the findings of this study are available from the corresponding author upon reasonable request.
